# High prevalence of self‐reported autism spectrum disorder in the Propionic Acidemia Registry

**DOI:** 10.1002/jmd2.12083

**Published:** 2019-12-10

**Authors:** Maria L. Cotrina, Sindy Ferreiras, Patricia Schneider

**Affiliations:** ^1^ Propionic Acidemia Foundation (PAF) Highland Park Illinois; ^2^ Department of Biology Queensborough Community College (QCC) Bayside New York

**Keywords:** ASD, autism, patient registry, propionic Acidemia, rare disease

## Abstract

Propionic Acidemia (PA) is characterized by the accumulation of propionic acid (PPA), its toxic derivatives, and ammonia. The disease causes multiorgan damage, especially in heart, pancreas, and brain; seizures and intellectual disability are often described. Some PA children also show autism spectrum disorders (ASD). In this study, we have compiled data from 62 individuals from the Propionic Acidemia International Patient Registry and compared it to the published literature on the prevalence of autism in PA. The PA registry shows a significant proportion of ASD diagnoses that is consistent with the combined prevalence reported in the literature. It also shows that ASD in PA is gender balanced and it is diagnosed at older ages (median age 8 years) than in the national registry for autism (median age 4.3 years), which raises the possibility, among others, of PA specific risk factors affecting the natural history of ASD. Data from patient registries provide valuable information on studying the mechanisms involved in a rare disease, although more outreach effort must be done to increase participation and consistency in data entry.

## 1 INTRODUCTION

Propionic Acidemia (PA) is an inborn error of metabolism caused by mutations in propionyl‐CoA carboxylase (PCC). The disease is characterized by the systemic accumulation of propionic acid (PPA) and its toxic derivatives. Children with PA are at high risk of developing low muscle tone, cardiomyopathy, neutropenia, pancreatitis, and ultimately death.^1^ PA also has neurological manifestations that include a high risk of seizures and stroke, and a wide range of chronic psychological and cognitive sequelae, with intellectual disability and language impairments being the most frequently reported.^2^

Some studies have reported an increased frequency of autism spectrum disorder (ASD)^3^ in children with PA.^4^, ^5^, ^6^ Similarly, there is evidence from studies in rats that high concentrations of PPA in the brain lead to abnormal behaviors, like delayed habituation to novelty in open‐field tasks^7^ or repetitive motor activity and hyperactivity.^8^ The relevance of this observation to PA is unclear given differences in methodology and exposure to PPA in addition to differential PCC enzyme activity, which make comparisons nearly impossible.

Despite these evidences, the true prevalence of ASD in PA and other inborn errors of metabolism has been difficult to assess given the high variability in the number of affected patients, in the testing tools reported in the literature, in the large number of comorbid factors that affect PA and, above all, in the heterogeneity in testing protocols. Moreover, the changing diagnostic criteria for ASD in the various versions of the Diagnostic and statistical manual of mental disorders (DSM, American Psychiatric Association) has also made it difficult to obtain reliable estimates of the prevalence of this condition in PA.

In this study, we collected information from the PA International Patient Registry maintained by the PA Foundation and compared it to the published prevalence of ASD in PA to obtain an improved estimate of the prevalence of this condition in this patient population.

## 2 METHODS

Data from PA and UCD‐affected patients were collected from the UCD/PA International Patient Registries, created in 2012, and maintained by the Propionic Acidemia Foundation (IL) and the National Urea Cycle Disorders Foundation (CA), respectively. All data were obtained in accordance with IRB‐approved protocols of the UCD/PA Registries. All registry entries are self‐reported either by the patients themselves, or by the parents/guardians in the case of a minor or a cognitively impaired person. Information is collected through a web‐based questionnaire consisting of 52 questions related to initial diagnosis and medical history of which a subset related to hospitalizations, hyperammonemia, cardiac, developmental or neurological diagnoses (including ASD, seizures, intellectual disability, stroke, basal ganglia damage, communication/language disorder) was included. For ASD, the registry had three possible answers: “Asperger's,” “Pervasive developmental disorder ‐not otherwise specified (PDD‐NOS)” and “Autism spectrum.” A positive answer to any of these entries was considered as a single instance of the self‐reported diagnosis of autism spectrum disorder. In each category, all the answers were structured: either with Yes/No type of answers or a list of possible answers from which the last option (“other”) is free text to capture an event or diagnosis not specified in the set of possible answers. For questions related to diagnoses, the questions required that the parent/caregiver reported only if the affected individual had been diagnosed by a medical professional about each specific condition.

For this manuscript, we have used the subset of questions that are common to both the UCD and the PA registries.

To calculate the percentage of cases for each diagnosis we divided the number of positive answers by the number of responders to that particular question. To compare the rates of ASD diagnosis between PA and UCD, we used Fisher's exact test at a level of confidence of 0.05.

To obtain an estimate of the published prevalence of ASD in PA, we carried out a literature search in PubMed using the keywords “autism/ASD and PA,” “autism/ASD and IEMs,” “cognitive delays and PA,” “developmental delays and PA,” “language impairment and PA.” For other IEMs, we performed a similar search including the terms “IEMs” (Inborn Error of Metabolisms), “UCDs” or “OAs” (Organic Acidemias). Inclusion criteria were set for any study referring to the neurological manifestations in propionic acidemia, UCDs or any of the other OAs or IEMs (prevalence of autism/ASD diagnoses, neurological manifestations in the form of developmental and cognitive delays, repetitive behaviors, communication/language impairments, stroke, basal‐ganglia damage).

## 3 RESULTS

### 3.1 High prevalence of self‐reported ASD in the registry

The PA international registry currently contains data from 104 participant families of which 62 (48 US; 13 international) self‐reported information related to neurological and developmental impairments in an affected family member and were used in this study. Table 1 summarizes the demographics of this cohort together with data on the manifestation of neurological and developmental disorders, including ASD, and its relationship with other comorbidities like hyperammonemia (51/52, 98%), epilepsy/seizures (14/47, 32%), and damage to other organs like cardiomyopathy (4/49, 8%) or optic‐nerve atrophy (4/46, 9%). The PA registry shows a substantial proportion of ASD diagnoses (10/48, 21%) that is consistent with the combined prevalence reported in the surveyed studies for PA (22%, Table S1).

**Table 1:** Characteristics of the population from the international PAF and UCDs registries

	PAF registry	PA literature	UCD registry	UCD literature
Number (male, female)	62 (27, 35)	85, 69 (43 unknown)	35, 53	
Age survey—median (range)	6 (1‐38)	3 months‐33 years	9 (1‐70)	
Age diagnoses	45/62 (73%) (under 1 month) 10/62 (16%) (1‐12 months) 6/62 (10%) (>1 year) 1/62 (1%) (dead before dx)	Birth‐20 years	41/82 (50%) 10/82 (12%) 31/82 (38%)	
High ammonia	2/52 (4%) (<100) 15/52 (29%) (100‐299) 8/52 (15%) (300‐499) 12/52 (23%) (500‐1000) 9/52 (17%) (>1200) 6/52 (12%) (Unsure)	18/24 (75%) (>80)^a^ 6/24 (25%) (>1000)	8/66(12%) 16/66 (24%) 16/66 (24%) 10/66 (15%) 16/66 (24%)	
Age highest ammonia	32/57 (59%) (first week) 12/57 (21%) (8 days‐6 months) 3/57 (5%) (6‐12 months) 7/57 (12%) (1‐2 years) 0 (> 2 years) 3/57 (5%) (Unsure)	‐	32/71 (45%) 3/71 (4%) 5/71 (7%) 5/71 (7%) 25/71 (35%)	
Developmental delays	36/53 (68%)	‐	23/59 (39%)	59‐100% (Jamiolkowski 2016)^9^
Cognitive impairment	18/46 (39%)	117/157 (74%)	7/56 (13%)	56‐79% (Gropman 2004)^10^ 55% (Serrano 2010)^11^ 17% (Waisbren 2016)^12^ 43% (Jamiolkowski 2016)^9^
Slow processing speed	10/43 (23%)	‐	10/54 (19%)	
Communication disorder	18/47 (38%)	111/168 (66%)	9/56 (16%)	11% (Serrano 2010)^11^
Attention deficit disorder	2/46 (4%)	26/132 (20%)	4/54 (7%)	29% (Jamiolkowski 2016)^9^
Attention‐deficit hyperactivity disorder	3/44 (7%)		5/57 (9%)	33% (Serrano 2010)^11^
Atypical social skills	12/49 (24%)	‐	5/55 (9%)	
Autism spectrum disorder (autistic behavior in^e^)	10/48 (21%)*	18/81 (22%)	3/49 (4%)	11% (Serrano 2010)^11^ 10% (Waisbren, 2016)^12^ >15% (Jamiolkowski 2016)^9^
Asperger's	1/45 (2%)	‐	1/51 (2%)	
Epilepsy/seizures	14/47 (32%)	38/84 (59%)	11/56 (20%)	
Stroke	3/46 (7%)	17/134 (13%)	0/51 (0%)	
Basal Ganglia damage	5/41 (12%)	18/56 (32%)	2/52 (4%)	
Cardiomyopathy	4/49 (8%)	15/109 (14%)^b^	n/a	
Long QT	3/49 (6%)	11/56 (22%)^c^	n/a	
Optic nerve damage	4/46 (9%)	6/55 (14%)^d^	1/52 (2%)	

*Statistically different from the incidence in UCD using Fisher's exact test (*P* < .05).

a Rafique et al.^13^

b Grunet et al.^14^ and Pena and Burton.^6^

c Grunet et al.^14^

d Pena and Burton.^6^

e Jamiolkowski et al.^9^

### 3.2 Comorbid conditions in the ASD‐diagnosed individuals in the PA International Patient Registry

We next examined the comorbid conditions present in the PA patients who were diagnosed with ASD as reported in the PA registry, and the relationship with their metabolic stability and other neurological and developmental manifestations (Table 2). Most subjects who received an ASD diagnosis have developmental delays (9/10, 90%), intellectual disability (7/10, 70%), and cardiac abnormalities (7/10, 70%). However, very few have basal‐ganglia damage (1/10, 10%) or stroke (1/10, 10%). Interestingly, the gender of the patients with an ASD diagnosis is balanced (five male and five female).

**Table 2:** Characteristics of PA individuals diagnosed with ASD in the PAF International Registry and comparison with comorbidities described for Autism Diagnoses

	Number (%)	aCDC 2018 number (%)
Number (male, female)	5 (50), 5 (50)	4920 (81, 19)
Median age at Autism diagnoses	8.0	4.3 (median)
Age diagnoses ≤ 8 years	6 (60)	
Age diagnoses ≥ 9 years	4 (40)	
Autism spectrum disorder	8 (80)	1810 (48)
Asperger's	1 (10)	238 (6.3)
PDD‐NOS (pervasive developmental disorder‐not otherwise specified)	1 (10)	1746 (46)
High ammonia	1 (10) <100 3 (30) 100‐500 2 (20) 600‐1000 2 (20) >1000 2 (20) unsure	‐
Age highest ammonia	7 (78) < 2 weeks old 1 (11) (1‐3 months) 1 (11) (1‐2 years)	‐
Developmental delays	9 (90)	‐
Intellectual disability	7 (70)	1151/3714 (31)
Slow processing speed	4 (40)	‐
Attention deficit disorder	1 (10)	‐
Attention deficit hyperactivity disorder	1 (10)	‐
Epilepsy/seizures	5 (50)	‐
Stroke	1 (10)	‐
Basal Ganglia damage	1 (10)	‐
Cardiac disorders (total)	7 (70)	‐
Cardiomyopathy	0	
Long QT	2 (20)	
Other cardiac	5 (50)	
Optic nerve atrophy	2 (20)	‐
Psychiatric disorders	0	‐
Gastro intestinal disorders	‐	‐

a Baio et al^15^ (Center for Disease Control—CDC‐report, 2018).

We also observed that PA patients were substantially older at age of ASD diagnosis (40% were 9 years of age or older at time of diagnosis with a median age of 12.5 years) than children in the general population (4.3 years old at age of diagnosis), even though PA children tend to be carefully monitored since birth.

### 3.3 The rate of ASD diagnosis in the PA registry is higher than in the UCD registry

To explore whether propionic acidemia is a risk factor for the prevalence of ASD, we compared the data from the PA registry to data reported in the UCD International Patient Registry because, similar to the PA patients, UCD patients typically exhibit hyperammonemia. As seen in Table 1, only 4% (3/49) of the patients in the UCD registry reported a diagnosis of ASD (statistically different using Fisher's exact test, *P* < .05).

## 4 DISCUSSION

### 4.1 The PA population is at an increased risk to be diagnosed with ASD

The data collected from the PA registry shows that about 21% of the PA population is diagnosed with ASD at some point in their lives and that this number is consistent with both the PA registry and the literature.

From this cohort we observed (a) that the age of diagnosis of ASD in PA obtained from the registry data is greater than the one reported in the national data, (b) that patients diagnosed with ASD often have concomitant intellectual impairment, as opposed to stroke or basal‐ganglia damage, and (c) that there was no higher prevalence of diagnoses in males over females, a difference from the known prevalence of ASD diagnoses in the general population, which tend to affect more boys than girls (ratio 4:1^15^). Although we found that the prevalence of ASD in the UCD registry affected girls over boys 2:1, potentially related to the frequency of ornithine transcarbamylase deficiency among females in the UCD population, the low number of cases reported prevents us from deriving any meaningful conclusion in the UCD population.

Consistent with de la Batie et al^4^ and Witters et al,^5^ ASD did not appear to be more frequent in patients with higher NH3 or strokes/basal‐ganglia damage in this patient population.

de la Batie et al^4^ also reported similar rates of autism in older children, equally distributed between male and female patients. In contrast, Witters et al^5^ found a correlation between sex, ASD (higher proportion in males than females), and genotype (higher frequency in patients with PCCB mutations), although no correlation with metabolic imbalance either.

Importantly, none of these reports found association between ASD and higher levels of 3‐hydroxypropionate (3‐OHP) in urine either. This is in contrast to the association found at early ages between higher 3‐OHP levels and intellectual disability.^4^

When we compared ASD prevalence in PA vs the general population, we found a much higher prevalence of ASD in PA (21% compared to 1.7% in the general population (1 in 59^15^), indicating that a diagnosis of PA is a risk factor to develop ASD and consistent with an increased risk of ASD in patients affected by other monogenic syndromes, like Fragile‐X‐syndrome, Rett syndrome or Duchenne muscular dystrophy.^16^

Our data raise the possibility that the quantitative exposure to high PPA levels in brain over time may be more relevant for an ASD diagnosis in PA patients than the intensity of the acute insult per se. In this regard, Waisbren et al^17^ also suggested that the number of metabolic crises in UCDs is not as closely associated with the neuropsychological outcomes as the number of years of exposure to abnormal levels of toxic metabolites.

It remains also possible that the increased diagnoses of ASD in older children in the registry is due to the implementation of new guidelines for diagnoses based on the DSM‐5, which came into effect in 2013. However, de la Batie et al also reported a similar observation in his European cohort utilizing the ADI‐R and ADOS‐2 instruments for autism diagnoses, tests implemented before 2013. If anything, the new DSM‐5 guidelines are more likely to exclude children from an ASD diagnosis than before.^15^

### 4.2 Comorbid conditions in the PA International Registry

Interestingly, the frequency of comorbid conditions such as intellectual disability epilepsy/seizures or cardiomyopathy is lower in the PA registry. A possible explanation for this discrepancy could be the median age of the registry participants (6 years‐old) compared to the age of the patients reported in the literature. It is possible that a younger cohort with earlier diagnoses (73% of the registry has been diagnosed under 1 month of age) means that less time has passed to observe the chronic consequences of the disease. Improved dietary management may also result in less comorbid conditions in this younger population. Self‐reported bias and the relatively small number of participants in the registry can also be added as other confounding factors.

It is expected that a higher participation in the registry will unmask the true prevalence of these comorbid conditions in PA.

### 4.3 The PA affected population show higher rates of ASD diagnosis than UCD population based on the registry data

The UCD International Registry provided a valuable comparative tool for the prevalence of ASD in other IEMs. Unfortunately, data from the UCD literature proved to be complex to interpret because the reports compile information from eight different UCD subtypes, each with early and late presentations, and with clear variations in neurological outcomes. It is worth mentioning that some of the outcomes reported tend to improve with treatments that diminish brain ammonia levels.^10^, ^18^ This is an important difference with the PA population where, even with stable, low levels of ammonia, there appeared to be a progression to ASD diagnosis as the children grew older.

### 4.4 Validity of the International PA and UCD Patient Registries

We have shown that the two registries utilized in this work have provided valuable information on the prevalence of ASD in a rare disease, but our work also highlights the complexity in evaluating a neurological disease like ASD in the PA population. There are limitations imposed by interobserver variation and more accurate and precise methodologies for both testing and comparing across studies are necessary.

Improvements can also be made to further increase the value of the patient registries: a stronger outreach effort must be done to involve families in the process of registry participation and families need to be empowered with clearer information about their child's diagnosis which, in turn, will increase accuracy in data entry in the databases.

Independently of the above caveats, the registries provide the unique capability to follow the same cohort over time as an initial and inexpensive source for longitudinal data.

## CONFLICT OF INTEREST

The authors declare that they have no conflict of interest.

## AUTHOR CONTRIBUTIONS

M.L.C. and P.S. conceived the idea, designed and supervised the project. M.L.C. and S.F. carried out all the investigation. M.L.C., P.S., and S.F. discussed the results and contributed to the final manuscript, written by M.L.C.

## ETHICAL APPROVAL STATEMENT

All procedures followed were in accordance with the ethical standards of the responsible committee on human experimentation (institutional and national) and with the Helsinki Declaration of 1975, as revised in 2000 (5). Informed consent was obtained from all patients for being included in the study. All data were obtained in accordance with IRB‐approved protocols of the UCD/PA Registries. No animals were used for this study.

## PATIENT CONSENT

All data presented in this manuscript are deidentified so no personal information about the patients was used. All patients signed an informed consent form as part of the requirement for participation in the registries.

## Supporting information


**Supplemental Table S1** Neurological and developmental sequelae of PA affected patients − prevalence of autism/ASD − Number (%)

## Data Availability

All original data utilized in this report is available from the Urea Cycle Disorder/Propionic Acidemia International Patient Registry upon request. The rest of the data comes from published literature.
