# Supplementary material for: High prevalence of self‐reported autism spectrum disorder in the Propionic Acidemia Registry
Source: JIMD Rep. 2019 Dec 10;51(1):70–5. doi: 10.1002/jmd2.12083 (PMC7012741; doi:10.1002/jmd2.12083)
Supplement: Supplementary file 1 — Supplemental Table S1 Neurological and developmental sequelae of PA affected patients − prevalence of autism/ASD − Number (%) [file JMD2-51-70-s001.docx]

**Supplemental Table 1.** **Neurological and developmental sequelae of PA affected patients – prevalence of autism/ASD – Number (%)**

| **Reference** | **Number**  **(Male, Female)** | **Age PA**  **diagnoses** | **Age evaluation** | **Cognitive Impairment** | **Language delays** | **Repetitive behavior** | **Attention deficit** | **Seizures** | **BasalGanglia/ Metabolic Stroke** | | **Autism/ASD diagnosis** |
| --- | --- | --- | --- | --- | --- | --- | --- | --- | --- | --- | --- |
| North et al. 1995 | 3, 3 | birth-6mo | 22 mo-13y | 6/6 (100) | 6/6 (100) | ---- | 1/6 (17) | ---- | ---- | | ---- |
| Nyhan et al. 1999 | 2, 0 | 7 mo-20y | 8-20y | 2/2 (100) | 2/2 (100) | ---- | ---- | 2/2 (100) | 2/2 (100) | ---- | ---- |
| Al-Owain et al. 2012 | 1 | birth | 1 d | 1/1 (100) | 1/1 (100) | 1/1 (100) | 1/1 (100) | ---- | ---- | ---- | 1/1 (100) |
| Pena et al. 2012 | 22, 36 | NBS-5y | 3mo-33y | 41/57 (72) | 40/53 (75) | ---- | 8/53 (15) | 24/58 (41) | ---- | 10/55 (18) | 5/53 (9) |
| Rafique M. 2013 | 16, 8 | Birth-13 mo | ---- | 18/24 (75) | 15/24 (62) | ---- | ---- | 12/24 (50) | ---- | 2/24 (8) | ---- |
| Grunert et al. 2013 | 35, 20 | 1d - 8 y | 5 d - 18 y | 30/40 (75) | 30/55 (55) | ---- | 11/48 (23) | ---- | 5/26  (19) | 5/55  (9) | ---- |
| Nizon et al., 2013 | 24 | Birth-  >1month | 1-18 y | 14/24 (61) | ---- | ---- | 5/24 (21) | ---- | 9/16  (56) | ---- | ---- |
| Witters et al. 2016 | 7, 1 | NBS-3y | 20 mo-6y | 6/8 (75) | 8/8 (100)_ | 3/8 (38) | ---- | ---- | 2/12  (17) | ---- | 8/8 (100) |
| De la Batie et al. 2018 | 19 | Birth->1month | 2 - 25 y | 13/19 (68) | 9/19 (47)* | ---- | ---- | ---- | ---- |  | 4/19 (21) |
| **TOTAL** |  |  |  | **117/157 (74)** | **111/168 (66)** | **4/9 (44)** | **26/132 (20)** | **38/84 (59)** | **18/56(32)** | **17/134**  **(13)** | **18/81 (22)** |

*In de la Batie et al., 5 individuals are reported to have social communication difficulties that we group as “language delays”

**In Nyhan et al. 1999 and Nizon et al.2013, there is no report of stroke incidence but of neurological abnormalities with basal ganglia involvement. In this table we have grouped the numbers reported of basal ganglia lesions under the category of metabolic stroke.

**Additional references for Supplemental Table 1:**

Al-Owain M, Kaya N, Al-Shamrani H, et al. 2013. Autism Spectrum Disorder in a child with Propionic Acidemia. JIMD Rep. 7:63-66. Doi:10.1007/8904_2012_143.

Nizon M, Ottolenghi C, Valayannopoulos V, et al. 2013. Long-term neurological outcome of a cohort of 80 patients with classical organic acidurias. Orphanet J Rare Dis 8: 148.

North KN, Korson MS, Gopal YR, et al. 1995. Neonata-onset propionic acidemia: neurologic and developmental profiles, and implications for management. J Pediatr 126, 916-922.

Nyhan WL, Bay C, Webb Beyer E, et al. 1999. Neurologic nonmetabolic presentation of propionic acidemia. Arch Neurol. 56, 1143-1147
